# Supplementary material for: Coffee consumption is associated with later age-at-onset of Parkinson’s disease
Source: medRxiv. 2025 Feb 10:2025.02.07.25321819. Preprint. [Version 1] doi: 10.1101/2025.02.07.25321819 (PMC11844581; doi:10.1101/2025.02.07.25321819)
Supplement: Supplement 1 — Supplementary Table 1. Cohorts included in the polygenic risk score analysis of caffeine consumption. Supplementary Table 2. Significant independent SNPs from coffee consumption GWAS (exposure) Supplementary Table 3. Heterogeneity tests and tests for directional horizontal pleiotropy between coffee consumption and PD risk, AAO and progression Supplementary Table 4. Genetic correlation between coffee consumption and PD risk and AAO. Supplementary Table 5. PRS of caffeine consumption in PD risk and AAO [file media-1.docx]

**Supplementary Table 1.** **Cohorts included in the polygenic risk score analysis of caffeine consumption.**

| Cohort | N total | Case | Control | Male, N (%) |
| --- | --- | --- | --- | --- |
| APDGC | 923 | 621 | 302 | 0.65 |
| McGill | 3651 | 2381 | 1270 | 0.41 |
| PPMI | 581 | 417 | 164 | 0.67 |
| IPDGC | 10799 | 5229 | 5480 | 0.59 |
| NGRC | 3940 | 1972 | 1968 | 0.53 |
| NIND | 1686 | 896 | 790 | 0.51 |
| AMP-PD | 5555 | 1963 | 3092 | 0.50 |

APDGC - Autopsy-Confirmed Parkinson Disease GWAS Consortium, McGill - McGill University Parkinson’s Cohort, PPMI - Parkinson's Progression Markers Initiative, IPDGC - International Parkinson Disease Genomics Consortium, NGRC - NeuroGenetics Research Consortium, NINDS - National Institute of Neurological Disorders and Stroke Repository Parkinson’s Disease Collection, AMP-PD - Accelerating Medicines Partnership Parkinson's disease

**Supplementary Table 2.** **Significant independent SNPs from Coffee consumption GWAS (exposure).**

| rsnumbers | MR with PD AAO* | Chr-position HG19 | Coffee consumption, P | PD AAO, P |
| --- | --- | --- | --- | --- |
| rs395815 | excluded | chr8:109156532 | 3.2e-09 | 0.67 |
| rs4410790 | included | chr7:17284577 | 1.9e-132 | 0.51 |
| rs2236955 | excluded | chr3:50429876 | 4.2e-08 | 0.45 |
| rs13387939 | included | chr2:637498 | 2.7e-13 | 0.79 |
| rs7224815 | excluded | chr17:17845800 | 5.7e-09 | 0.56 |
| rs4357572 | excluded | chr1:50576710 | 5.9e-11 | 0.91 |
| rs34060476 | included | chr7:73037956 | 1.2e-22 | 0.77 |
| rs2465054 | excluded | chr6:51174232 | 9.2e-09 | 0.48 |
| rs1422191 | excluded | chr5:87949158 | 4.1e-09 | 0.29 |
| rs55754437 | included | chr4:2933031 | 1.1e-08 | 0.90 |
| rs17004922 | excluded | chr22:24844948 | 4.7e-08 | 0.66 |
| rs6062357 | included | chr20:62892739 | 4.0e-11 | 0.79 |
| rs6063085 | excluded | chr20:45840459 | 4.8e-09 | 0.70 |
| rs13387939 | included | chr2:27730940 | 6.2e-17 | 0.77 |
| rs56113850 | excluded | chr19:41353107 | 3.2e-15 | 0.04 |
| rs66723169 | excluded | chr18:57808978 | 9.1e-19 | 0.35 |
| rs4092465 | excluded | chr18:55080437 | 3.3e-08 | 0.73 |
| rs62064918 | excluded | chr17:46155786 | 8.6e-09 | 0.08 |
| rs8056750 | excluded | chr16:70927078 | 1.5e-08 | 0.78 |
| rs17817964 | excluded | chr16:53828066 | 6.1e-17 | 0.28 |
| rs117968677 | excluded | chr15:75174251 | 5.1e-11 | 0.19 |
| rs2472297 | included | chr15:75027880 | 3.3e-168 | 0.57 |
| rs1057868 | included | chr7:75615006 | 7.6e-34 | 0.65 |
| rs2763981 | included | chr6:31840021 | 5.1e-10 | 0.68 |
| rs34190000 | excluded | chr5:7381260 | 6.5e-10 | 0.26 |
| rs2521501 | included | chr15:91437388 | 1.4e-08 | 0.63 |
| rs61928609 | included | chr12:11316437 | 1.2e-11 | 0.75 |
| rs539515 | included | chr1:177889025 | 2.7e-09 | 0.88 |

*Some SNPs were excluded from the Mendelian Randomization (MR) analysis on Parkinson’s disease (PD) age at onset (AAO) because they showed high pleiotropy as identified by MR-PRESSO.

**Supplementary Table 3.** **Heterogeneity tests and tests for directional horizontal pleiotropy between coffee consumption and PD risk, AAO and progression**

| Outcome | Heterogeneity tests | | | | | | Test for directional horizontal pleiotropy | | | |
| --- | --- | --- | --- | --- | --- | --- | --- | --- | --- | --- |
|  | MR Egger | | | Inverse variance weighted | | | egger_intercept | se | pval | MR-PRESSO global |
|  | Q | Q_df | Q_pval | Q | Q_df | Q_pval |  |  |  | pval |
| PD AAO | 18.289 | 10 | 0.051 | 18.35 | 11 | 0.074 | -0.003 | 0.017 | 0.851 | 0.202 |
| PD risk | 8.026 | 15 | 0.922 | 8.05 | 16 | 0.947 | 0.001 | 0.012 | 0.882 | 0.944 |
| UPDRS3 | 9.22 | 19 | 0.969 | 9.35 | 20 | 0.978 | 0.003 | 0.010 | 0.726 | 0.976 |
| MMSE | 0.236 | 2 | 0.889 | 0.25 | 3 | 0.970 | -0.005 | 0.051 | 0.928 | 0.976 |
| MoCa | 0.058 | 5 | 0.999 | 0.07 | 6 | 0.999 | 0.014 | 0.124 | 0.915 | 0.972 |
| Hyposomia | 9.224 | 19 | 0.970 | 9.35 | 20 | 0.979 | 0.004 | 0.010 | 0.726 | 0.974 |
| Sleep | 0.668 | 5 | 0.985 | 0.79 | 6 | 0.992 | -0.023 | 0.065 | 0.740 | 0.974 |

PD – Parkinson’s disease; AAO - Age at Onset; pval - p-value ; UPDRS3 - unified Parkinson's disease rating scale part 3; MMSE - Mini Mental State Examination; MoCA - Montreal Cognitive Assessment; OR - Odds ratio, L_CI95- low 95% confidence interval; U_CI95- upper 95% confidence interval

**Supplementary Table 4. Genetic correlation between coffee consumption and PD risk and AAO GWASs**

| **Outcome** | **rg** | **se** | **z** | **P** |
| --- | --- | --- | --- | --- |
| **PD risk** | 0.001 | 0.032 | 0.028 | 0.978 |
| **PD AAO** | -0.096 | 0.076 | -1.2592 | 0.208 |

PD – Parkinson’s disease; AAO – Age-at-onset; rg – genetic correlation coefficient; se – standard error.

**Supplementary Table 5. PRS of caffeine consumption in PD risk and AAO**

| PRS coffee consumption with PD risk | | | | |
| --- | --- | --- | --- | --- |
|  | OR | 95%-CI | %W(random) | p-value meta-analysis |
| Mcgill | 1.077 | [0.987;1.174] | 9.5 |  |
| PPMI | 1.026 | [0.845;1.247] | 1.9 |  |
| APDGC | 1.035 | [0.892;1.201] | 3.2 |  |
| IPDGC | 0.992 | [0.954;1.032] | 46.8 |  |
| NIND | 1.049 | [0.945;1.165] | 6.6 |  |
| NGRC | 1.048 | [0.974;1.128] | 13.4 |  |
| AMP PD | 1.023 | [0.968;1.096] | 18.5 |  |
| Meta-analysis Random effect model | 1.02 | [0.993;1.048] | | 0.147 |
| PRS coffee consumption with PD AAO | | | | |
|  | BETA | 95%-CI | %W(random) |  |
| Mcgill | -0.131 | [-0.556; 0.295] | 20.1 | p-value meta-analysis |
| PPMI | -0.086 | [-1.126; 0.953] | 3.4 |  |
| APDGC | 0.252 | [-0.423; 0.927] | 8 |  |
| IPDGC | -0.031 | [-0.378; 0.316] | 30.3 |  |
| NIND | 0.412 | [-0.328; 1.151] | 6.7 |  |
| NGRC | 0.368 | [-0.165; 0.901] | 12.8 |  |
| AMP PD | 0.141 | [-0.301; 0.583] | 18.6 |  |
| Meta-analysis Random effect model | 0.083 | [-0.1084; 0.2735] | | 0.397 |

OR – Odds Ratio, CI – Confidence Interval, PPMI – Parkinson's Progression Markers Initiative, APDGC – Autopsy-Confirmed Parkinson Disease GWAS Consortium, IPDGC – International Parkinson Disease Genomics Consortium, NINDS – National Institute of Neurological Disorders and Stroke Repository Parkinson's Disease Collection, NGRC – NeuroGenetics Research Consortium, AMP-PD - Accelerating Medicines Partnership Parkinson's disease
